# Post-operative complications of cholecystectomy: what the radiologist needs to know

**DOI:** 10.1007/s00261-024-04387-5

**Published:** 2024-06-28

**Authors:** Irfan Amir Kazi, M. Azfar Siddiqui, Nanda Deepa Thimmappa, Amr Abdelaziz, Ayman H. Gaballah, Ryan Davis, Eric Kimchi, Ghassan Hammoud, Kazi A. Syed, Ayesha Nasrullah

**Affiliations:** 1 Department of Radiology, University Hospital, University of Missouri, 1 Hospital Drive, Columbia, MO 65212 USA; 2 Department of Radiology, University of Missouri, Columbia, MO USA; 3 Department of Radiology, MD Anderson Cancer Center, University of Texas, Houston, TX USA; 4 Department of Surgical Oncology, University of Missouri, Columbia, MO USA; 5 Department of Gastroenterology, University of Missouri, Columbia, MO USA; 6 Medical Student, Kansas City University College of Osteopathic Medicine, Kansas, MO USA

**Keywords:** Cholecystectomy complications, Bile leak, Bile duct injury, Dropped gallstones

## Abstract

Cholecystectomy is one of the most performed surgical procedures. The safety of this surgery notwithstanding, the sheer volume of operations results in a notable incidence of post-cholecystectomy complications. Early and accurate diagnosis of such complications is essential for timely and effective management. Imaging techniques are critical for this purpose, aiding in distinguishing between expected postsurgical changes and true complications. This review highlights current knowledge on the indications for cholecystectomy, pertinent surgical anatomy and surgical technique, and the recognition of anatomical variants that may complicate surgery. The article also outlines the roles of various imaging modalities in identifying complications, the spectrum of possible postsurgical anatomical changes, and the implications of such findings. Furthermore, we explore the array of complications that can arise post-cholecystectomy, such as biliary system injuries, gallstone-related issues, vascular complications, and the formation of postsurgical collections. Radiologists should be adept at identifying normal and abnormal postoperative findings to guide patient management effectively.

**Graphical abstract:**

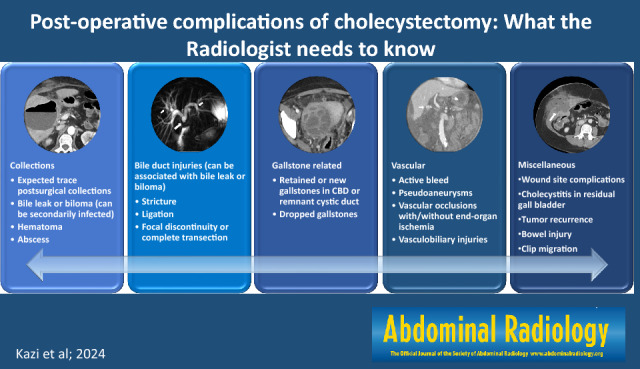

## Introduction

Cholecystectomy, the surgical removal of the gallbladder, is among the most frequently performed abdominal operations in the United States, with around three-quarters of a million procedures carried out each year [1, 2]. Its prevalence is largely due to the high incidence of gallstone disease and its associated complications, which are the primary indications for the surgery [2, 3]. The advent of laparoscopic techniques has revolutionized cholecystectomy, offering a minimally invasive approach that has become the standard of care. Despite the recognized safety and efficacy of laparoscopic cholecystectomy, its widespread practice inevitably leads to a spectrum of postoperative complications [2–4].

Timely and accurate identification of post-cholecystectomy complications is crucial, as it can significantly influence patient outcomes, reducing the morbidity associated with delayed diagnosis and treatment [5, 6]. Imaging is an indispensable tool providing a non-invasive means of differentiating between normal postoperative anatomy and pathology. Based on the indication, ultrasound and computed tomography (CT) are the primary imaging modalities used to evaluate postsurgical complications. CT is essential for the diagnosis of vascular complications. Magnetic resonance cholangiopancreatography (MRCP) is the imaging modality of choice for evaluating the biliary tree. Bile leak can be diagnosed on a radionuclide scan or on magnetic resonance imaging using hepatobiliary-specific contrast agents [7, 8].

This review article provides an overview of the common indications for cholecystectomy and the surgical anatomy underpinning the procedure. It describes the nuances of the surgical technique, with particular attention to the identification of anatomical variants that may predispose patients to complications. These variants play a pivotal role in the complexity and outcome of the surgical procedure. Additionally, we discuss the role of imaging in the postoperative setting, its utility in identifying complications, and the importance of distinguishing between normal postsurgical changes and complications.

### Common indications for cholecystectomy

Symptomatic gallstone disease is the most common indication for cholecystectomy. Other indications include acute cholecystitis, chronic cholecystitis, choledocholithiasis, gallstone pancreatitis, gall bladder polyps and masses, and biliary dyskinesia [9, 10].

### Relevant surgical anatomy and surgical technique

**Fig. 1:**
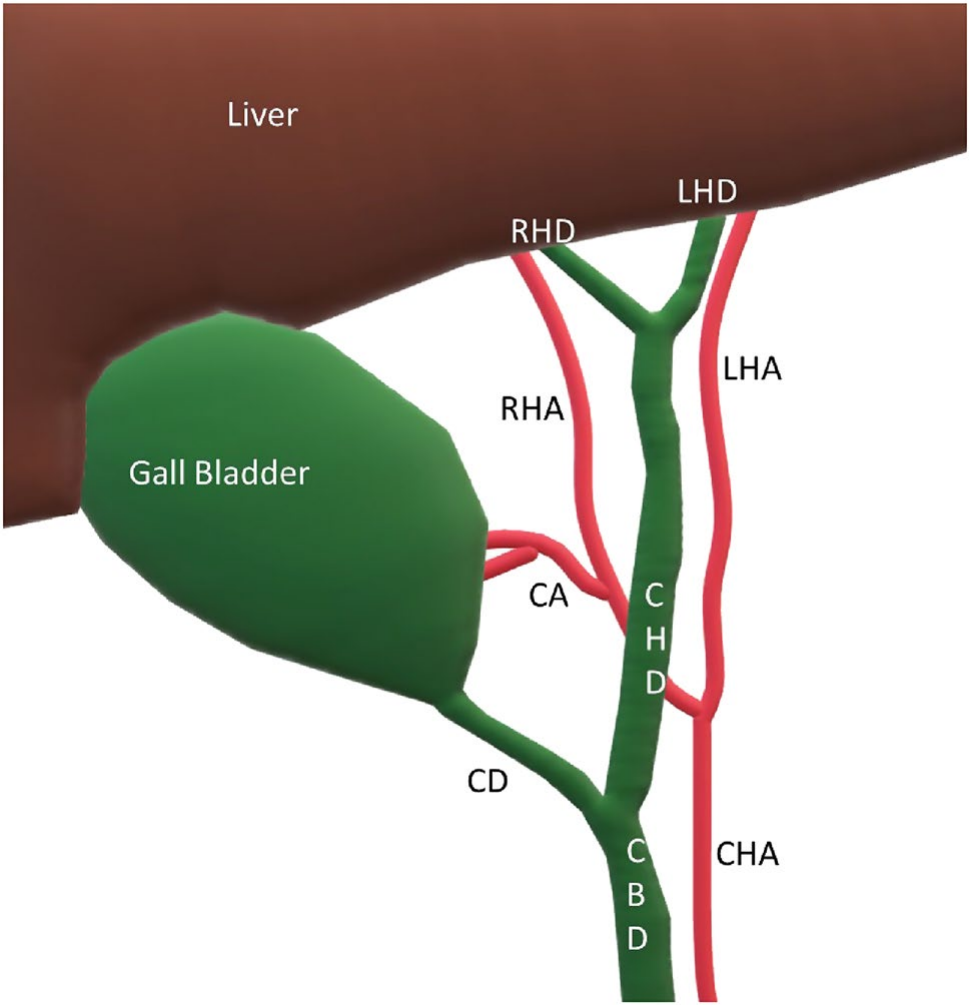
Surgical landmarks for cholecystectomy: Calot’s triangle bounded superiorly by the inferior surface of the liver, inferiorly by the cystic duct (CD), and on the left side by the bile duct (CBD & CHD). Important contents of the Calot’s triangle include the Right hepatic artery (RHA) and the cystic artery (CA), a branch of the right hepatic artery. The cystic artery is identified and ligated during surgery to prevent hemorrhage

The Calot's triangle (hepatocystic triangle) serves as an anatomical landmark during cholecystectomy. It is an imaginary triangle bordered on the left side by the common hepatic duct, inferiorly by the cystic duct, and superiorly by the inferior liver surface (Fig. 1). It contains the right hepatic artery and its branch, the cystic artery (Fig. 1), along with connective tissue and the cystic lymph node of Lund (the first lymph node of the gallbladder) [11]. Dissection in the hepatocystic triangle is performed to obtain the 'critical view of safety'. Misidentifying the normal anatomy and failing to recognize anatomical variants of significance can lead to surgical complications [12]. It is imperative to ensure that only two structures enter the gallbladder: the cystic duct and the cystic artery [3, 12]. The cystic artery and the cystic duct are ligated, and then the gallbladder is removed [3]. Intraoperative cholangiogram may be routinely performed by some surgeons [3]. Others may use it as a problem-solving tool if biliary anatomy is uncertain or there is suspicion of biliary injury or for evaluating possible choledocholithiasis [3, 12].

### Variants of significance in biliary and arterial anatomy

**Fig. 2:**
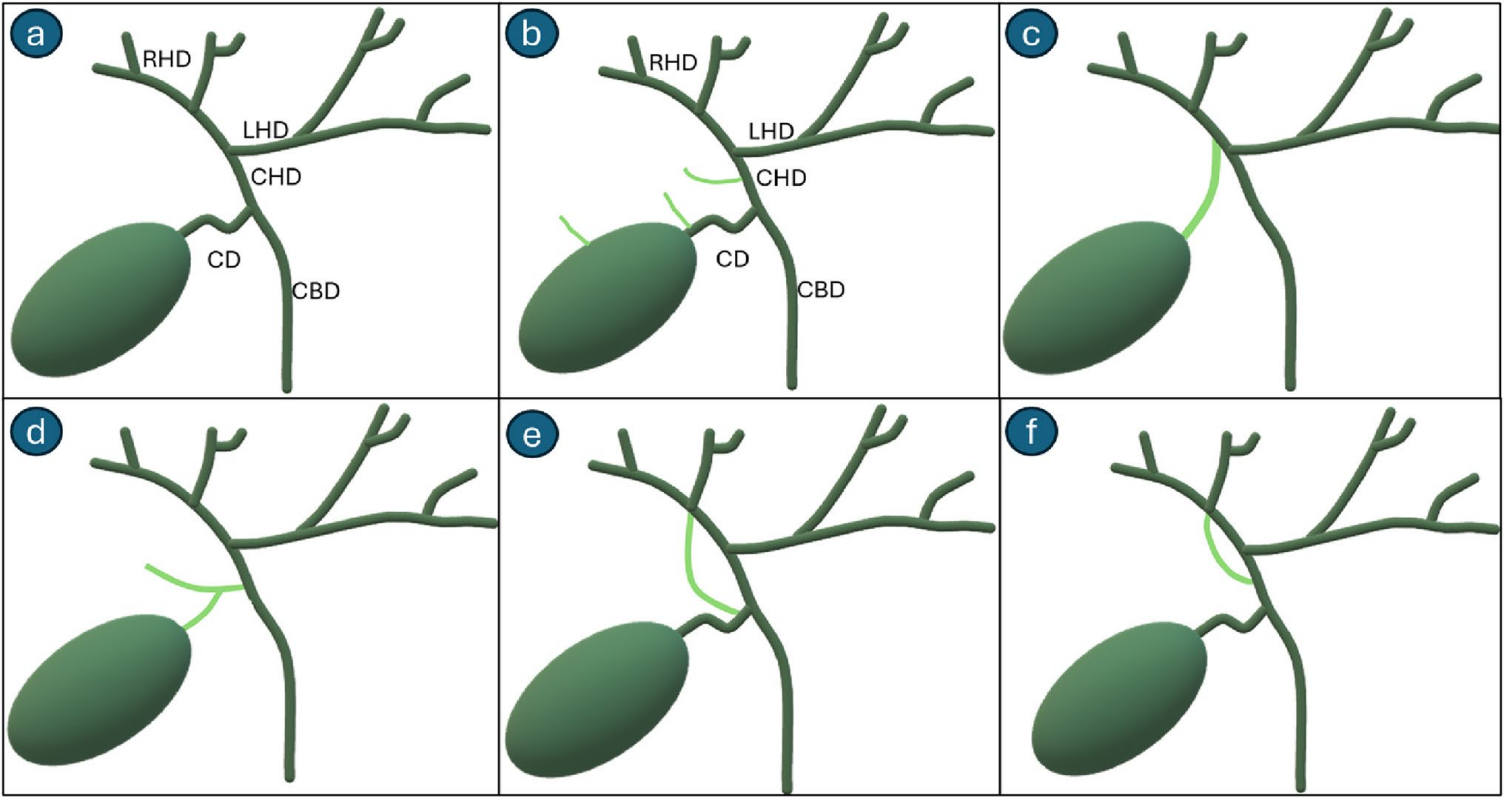
Common variants of the biliary tree in relation to cholecystectomy. **a** Normal biliary anatomy **b** Subvesical ducts of Luschka, accessory bile ducts, or aberrant bile ducts can drain into the extrahepatic biliary tree. **c** and **d** The cystic duct can drain into the right hepatic duct or into an accessory bile duct. **e** and **f** The right-sided bile ducts, especially the right posterior duct, can drain into the cystic duct or into the common duct. CBD: Common bile duct. *RHD* Right hepatic duct, *LHD* Left hepatic duct, *CHD* Common hepatic duct, *CD* Cystic duct

It is crucial to recognize variants in the biliary anatomy to avoid injury to the biliary system during the surgery. The Hartman's pouch, an anatomical variant, is a bulge that may be present in the neck of the gallbladder. If prominent, it can obscure the visualization of the cystic duct and the Calot's triangle [12]. Small bile ducts arising from the right lobe of the liver, the subvesical ducts (Ducts of Luschka), can drain into the extrahepatic bile ducts or into the gallbladder [13] (Fig. 2b). Accessory or aberrant bile ducts may drain into the right hepatic duct in the Calot's triangle (Fig. 2b). An aberrant bile duct is the only pathway of biliary drainage of the portion of the liver that it drains, while an accessory duct represents a duct providing an additional path for biliary drainage [14]. Significant variants in the anatomy of the cystic duct include low parallel insertion of the cystic duct into the common hepatic duct and a spiral configuration of the cystic duct traversing posterior to the common hepatic duct before its insertion on the left side of the common duct. Drainage of the cystic duct into the right hepatic duct (Fig. 2c), duplication of the cystic duct, short course of the cystic duct, or congenitally absent cystic duct are other rare variants of significance [11, 12, 15]. Accessory ducts seen during cholecystectomy generally drain the right lobe (Fig. 2b). They typically traverse the Calot’s triangle and drain into the common hepatic duct inferior to the confluence of the right and the left hepatic ducts. Rarely, the cystic duct may drain into an accessory duct [12] (Fig. 2d). The right posterior duct, a sectoral duct draining segments VI and VII, which commonly unites with the right anterior hepatic duct to form the right hepatic duct, may aberrantly drain into the common hepatic duct or the cystic duct [12, 14] (Fig. 2e and f). MRCP may help in the preoperative diagnosis of some of these abnormalities, while CT and ultrasound are not particularly helpful in diagnosing biliary anatomical variants.

Significant variants in the arterial vasculature include a replaced or accessory right hepatic artery originating from the superior mesenteric artery (10–22%) or common hepatic artery originating from the abdominal aorta (2%), which can have a variant course in the Calot’s triangle. If there is a tortuous course of the right hepatic artery in the Calot’s triangle, known as ‘Moynihan’s hump’, the right hepatic artery may be mistaken for a cystic artery. Occasionally, two cystic arteries may be present [11, 12]. Rarely, the cystic artery may arise from arteries other than the right hepatic artery [12].

### Other factors predisposing to surgical complications

In addition to the anatomical variants discussed above, impaired visualization and difficult dissection increase the risk of developing surgical complications. Inflammatory changes and adhesions/fibrosis in the surgical bed can obscure the anatomy, causing difficulty in surgery [16]. Obesity can lead to difficult dissection and impaired identification of anatomical landmarks and variants [2, 12]. Underlying liver disease can increase the risk of operative blood loss [17, 18].

### Surgical approaches and types of cholecystectomy

Cholecystectomy was traditionally performed via an open approach. Laparoscopic cholecystectomy, introduced in the 1980s, is the current standard and preferred method of cholecystectomy for symptomatic gallbladder stones due to the advantages of lower morbidity, faster recovery, less pain, and better cosmetic results when compared to open cholecystectomy. Laparoscopic cholecystectomy may be converted into an open procedure in 4 to 8% of the cases. Typically, a conversion to an open surgical approach is utilized in more challenging procedures where surgical landmarks are not clearly identifiable due to inflammation/scarring, anatomical variants requiring further clarity, or intraoperative complications necessitating an open procedure [2]. Robotic cholecystectomy is becoming increasingly popular. It has a similar rate of postsurgical complications when compared to the laparoscopic route. Its advantages include three-dimensional visualization and a lower conversion rate into an open procedure. The disadvantages include increased cost of the procedure and longer surgery time [19, 20].

**Fig. 3:**
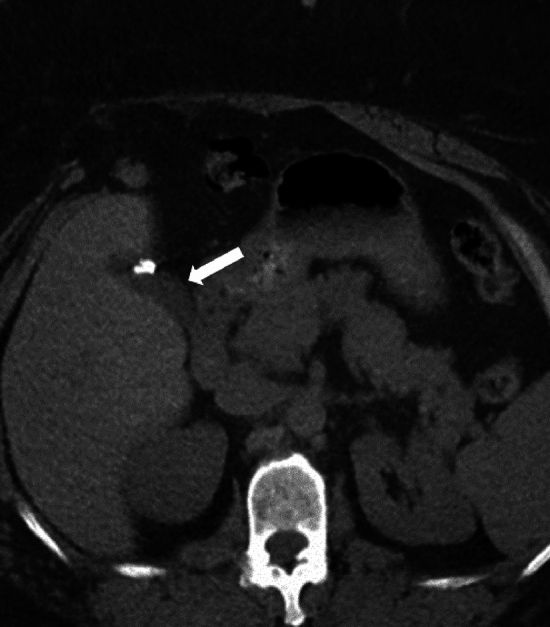
A 35-year-old female with subtotal reconstituting cholecystectomy (arrow) for symptomatic cholelithiasis. Complete cholecystectomy could not be performed as a critical view of safety could not be obtained during the surgery due to morbid obesity

During a cholecystectomy, the entire gallbladder is typically removed, termed as total cholecystectomy. However, if challenges arise during surgery that prevent the safe removal of the entire organ, a subtotal cholecystectomy may be performed. Types of subtotal cholecystectomy include subtotal fenestrating cholecystectomy and subtotal reconstituting cholecystectomy. In subtotal fenestrating cholecystectomy, a part of the gallbladder wall adherent to the liver is not removed, and the exposed mucosal surface is ablated, followed by ligation of the cystic duct. As a result, there is no residual gallbladder lumen, and it is not associated with an increased risk of cholecystolithiasis. However, there is an increased risk of developing a bile leak. In subtotal reconstituting cholecystectomy, the residual gallbladder is sutured to form a lumen, which is continuous with the cystic duct (Fig. 3). As there is a residual gallbladder lumen, potentially cholelithiasis and cholecystitis can occur [8, 21]. The spectrum of complications that can occur post-total cholecystectomy can also develop post-subtotal cholecystectomy.

Percutaneous cholecystostomy is a non-surgical alternative in patients who are at high risk for perioperative complications and can be performed as a stop-gap or as a definitive procedure. It is performed under imaging guidance and can utilize ultrasound, CT, and fluoroscopy, either individually or in combination. The gallbladder can be entered via either transhepatic or transperitoneal approaches. Common complications include catheter dislodgment, bile peritonitis, hemobilia, and gallbladder perforation [22, 23]. Endoscopic procedures may also be used to drain the gallbladder. Endoscopic transpapillary drainage of the gallbladder is a well-established procedure. Endoscopic ultrasound-guided transmural gallbladder drainage via the stomach or the duodenum using a lumen-apposing metal stent is also gaining popularity [22, 24, 25].

### Imaging modalities in the evaluation of postsurgical complications

Various imaging modalities can be used to evaluate postsurgical complications and should be tailored as per the indication. Ultrasound and Computed Tomography (CT) are the first imaging lines, depending upon the indication. Ultrasound is useful in assessing liver pathology, postsurgical collections, and evaluation for intrahepatic biliary dilatation. CT ​ provides excellent spatial resolution with fast acquisition time. It accurately depicts foci of air and calcifications. A single-phase CT study is typically performed in the portal venous phase after intravenous contrast administration. A multiphase CT study must be performed if there is suspicion of active bleeding. Magnetic resonance imaging (MRI) provides excellent soft tissue resolution, and magnetic resonance cholangiopancreatography (MRCP) is ideal for imaging the biliary tree and evaluating choledocholithiasis. MRI using hepatobiliary contrast helps diagnose bile leaks. MRI is limited by the long duration of examination and dependence on breath-holding and is suboptimal for the evaluation of air and calcifications. Hepatobiliary scintigraphy in the form of a hepatobiliary iminodiacetic acid (HIDA) scan can also help confirm a bile leak. A SPECT-CT study provides a better anatomical overview of the bile leak when compared to the planar HIDA scan and can be helpful in problem-solving. A percutaneous transhepatic cholangiogram (PTC) or an endoscopic retrograde cholangiopancreatography (ERCP) may be performed as a part of diagnostic work-up and interventional management of bile leaks. Angiography may be required to evaluate and manage vascular complications [8, 15, 26, 27].

### Expected postsurgical changes

**Fig. 4:**
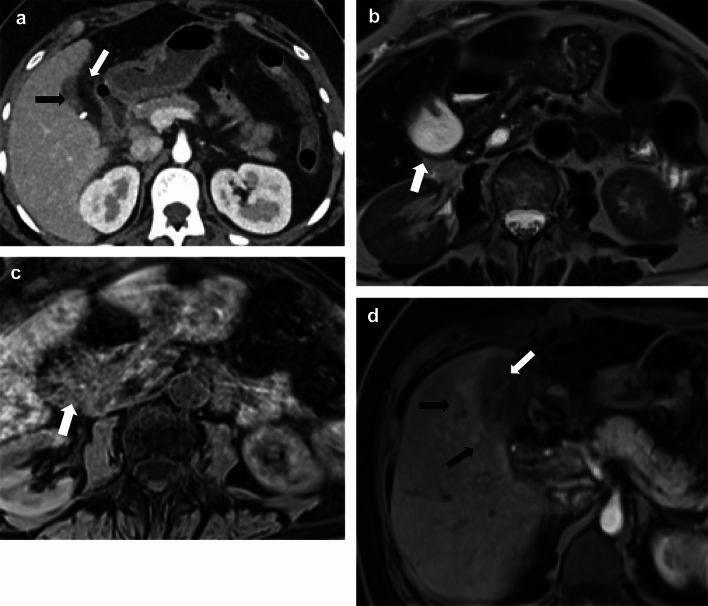
Expected changes post cholecystectomy. **a** A 27-year-old with abdominal pain post cholecystectomy. An axial CT image showing trace collection (black arrow) and fat stranding in the gallbladder fossa (white arrow), which are expected postsurgical changes. **b** and **c** A 75-year-old female with small postsurgical hematoma post-cholecystectomy. **b** Axial T2 and **c** Axial precontrast T1W images demonstrate a T2 hyperintense collection that contains intrinsic T1 signal suggestive of blood products (arrow). **d** A 72-year-old post-recent cholecystectomy. Axial T1W post-contrast image shows reactive hyperemia in the adjacent liver (black arrows). Expected trace collection and fat stranding in the gallbladder fossa (white arrow).

Small fluid collections can be seen in the gallbladder fossa and in the abdominal cavity (Fig. 4a). These collections can represent simple fluid or small postsurgical seromas or hematomas. If the collection has blood products within, it can show a high signal on the noncontrast T1-weighted image (Fig. 4 b and c). Although there is no specific timeframe that can be used to differentiate expected versus pathological collections, large collections and progressive increases in the size of the postsurgical collections should raise a concern for pathological collections, while expected postsurgical collections will stay stable or show progressive interval decrease in size [26]. Fat stranding can be seen in the region of the gallbladder fossa and at the incision site in the anterior abdominal wall [27]. Reactive inflammatory changes may be seen in the liver adjacent to the gallbladder fossa (Fig. 4d). Contrast-enhanced CT and MRI will demonstrate hyperenhancement when compared to the rest of the liver parenchyma.

**Fig. 5:**
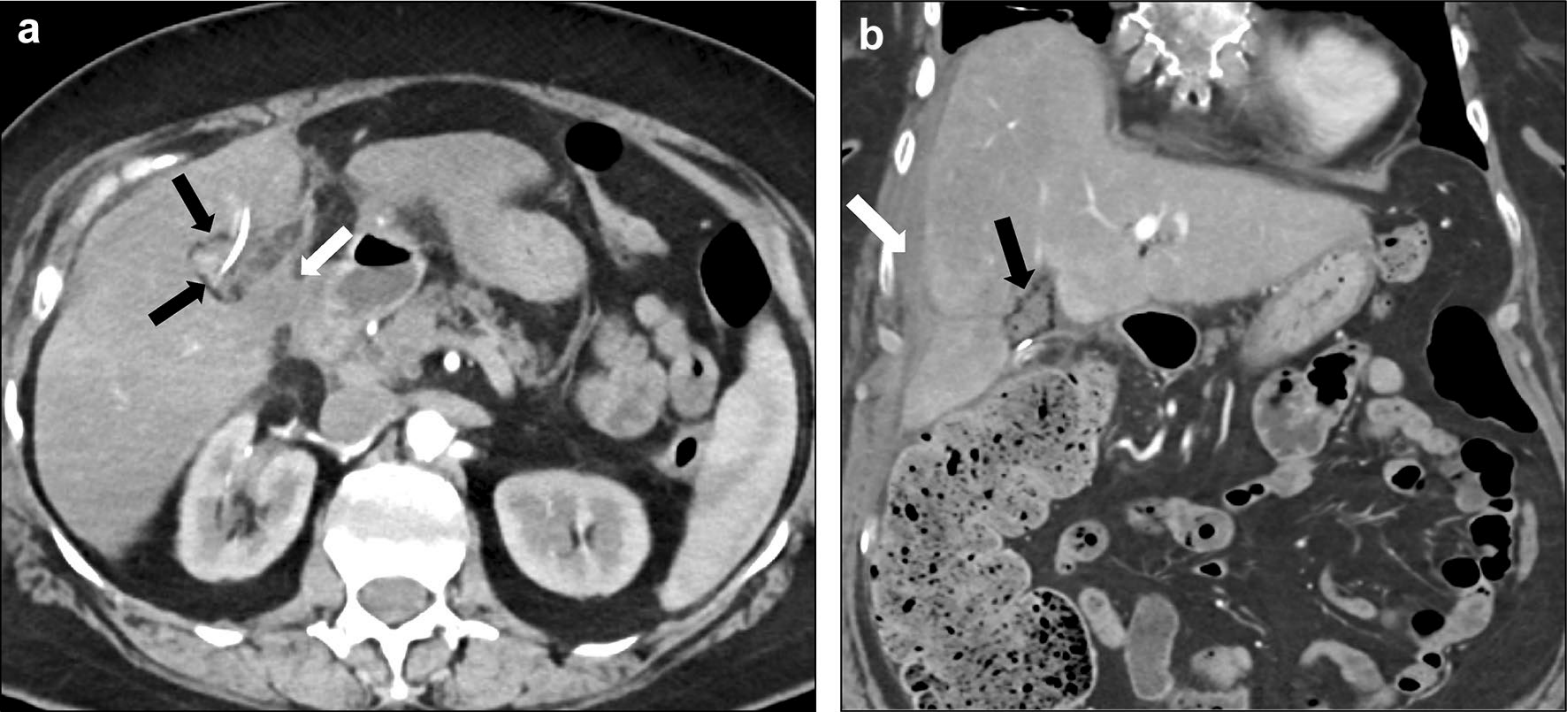
A 66-year-old woman who underwent cholecystectomy for acute calculus cholecystitis, with surgicel material in the gall bladder fossa. **a** and **b** Postoperative axial and coronal CECT images showing Surgicel material in the gallbladder fossa (black arrows) and a small volume high-density collection in the gallbladder fossa, and perihepatic region suggestive of postsurgical hematomas/seromas (white arrows)

Hemostatic material (Surgicel) may be left in the surgical bed and should not be mistaken for an abscess, hematoma, or retained surgical material (Fig. 5). Surgicel can have varied imaging appearances but is most commonly seen as focal air collections within a mixed attenuation collection/mass. Surgicel, a cellulose-based product, does not have a radiodense marker, while a retained surgical sponge generally has a radiodense marker. Reviewing surgical notes and discussing with the surgeon are helpful in such diagnostic dilemmas [27, 28].

Dilatation of the intrahepatic and extrahepatic biliary system can be seen post cholecystectomy, as the biliary system serves as a reservoir for bile after gallbladder removal. The common bile duct can progressively and physiologically dilate up to 10 mm post-cholecystectomy [29]. Prexisting dilatation may be seen in the setting of a choledochal cyst, If there is dilatation of the biliary tree, especially when associated with an obstructive pattern of liver function tests, an MRCP may be performed to rule out choledocholithiasis or an underlying mass. If the MRCP does not demonstrate an obstructing cause, the differential diagnosis includes a radiologically occult mass, ampullary stenosis, and sphincter of Oddi dysfunction versus expected postsurgical changes [27, 29].

### Postoperative complications

We have categorized post-cholecystectomy complications into A. Injuries to the biliary system, B. Gallstone-related complications, C. Vascular complications, D. Postoperative collections, and E. Miscellaneous complications. These complications can occur independently, sequentially, or simultaneously.

### Injuries to the biliary system

Although biliary injuries are rare, with an estimated incidence of 0.4–1.5%, they are of significant concern, leading to increased morbidity and mortality, along with possible long-term sequelae [30]. These injuries occur more frequently during laparoscopic surgeries than open cholecystectomies; the incidence post-open cholecystectomy ranges from 0.1–0.5%, while the reported incidence with laparoscopic procedures is 0.5–1.5% [31, 32].

Factors contributing to these injuries include incorrect identification of biliary anatomy, failure to recognize anatomical variants, imprecise cystic or bile duct ligation, dislodgment of surgical clips, or thermal damage from cautery. These can lead to partial or complete ductal discontinuity leading to bile leak, development of biliary stricture leading to biliary obstruction, or both [33]. The sources of bile leak include leak from a peripheral duct in the gallbladder fossa (duct of Luschka), the cystic duct stump, a partial injury to the side of a hepatic duct, or a transected hepatic or common bile duct. The risk of bile leak is higher for surgeries on severely inflamed gallbladders and in patients with subtotal cholecystectomies. Biliary stricture/obstruction can be either acute or chronic. Biliary injuries may be diagnosed during the procedure, in the immediate or late postoperative periods. Prompt diagnosis generally correlates with better outcomes [30, 33]. In the early postoperative period, such injuries may result in cholangitis and intraabdominal abscesses. Long-term effects of chronic biliary obstruction include biliary strictures, intrahepatic lithiasis, secondary biliary cirrhosis, and portal hypertension [6, 34].

**Fig. 6:**
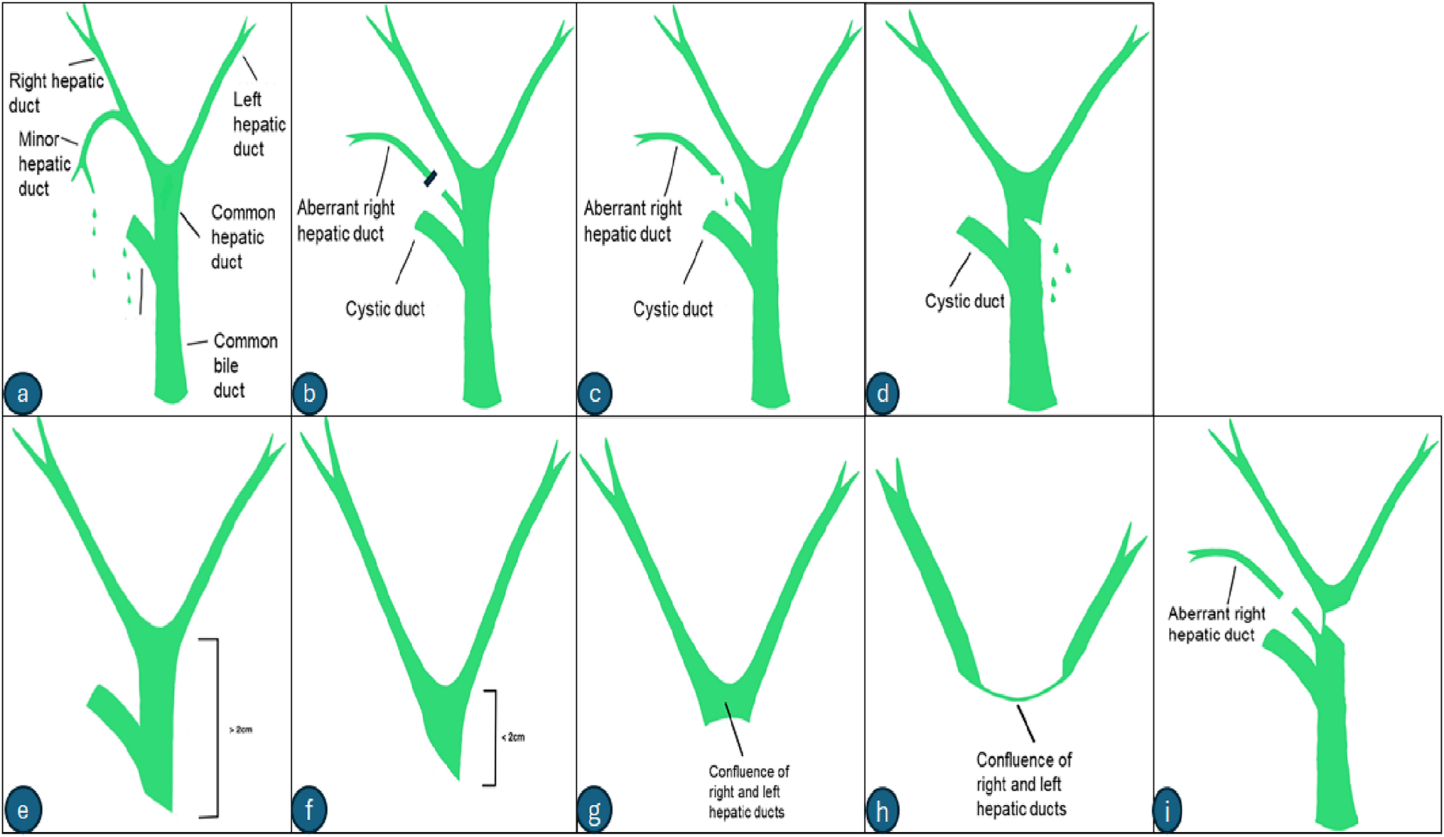
Bismuth and Strasberg classification of postcholecystectomy biliary injuries. **a** Strasberg type A injury, bile leak from the cystic duct or from small ducts in the gallbladder fossa. **b** Strasberg type B injury, occlusion of an aberrant right posterior hepatic duct. **c** Strasberg type C injury, transection of an aberrant right hepatic duct without ligation. **d** Strasberg type D injury, a lateral injury involving a major bile duct. **e** Strasberg type E1/Bismuth type 1 injury, injury to the common bile duct or the common hepatic duct > 2 cm from the hilar confluence. **f** Strasberg type E2/Bismuth type 2 injury, injury to the or the common hepatic duct < 2 cm from the hilar confluence. **g** Strasberg type E3/Bismuth type 3 injury, hilar biliary injury with intact confluence. **h** Strasberg type E4/Bismuth type 4 injury, hilar biliary injury with description of confluence. **i** Strasberg type E5/Bismuth type 5 injury, injury to an aberrant right hepatic duct with an injury to the common hepatic duct

Various classification systems have been proposed for classifying biliary injuries during cholecystectomy. Accurate diagnosis of the type and extent of injury helps decide the appropriate management. The Bismuth and the Strasberg classifications are discussed in the present article. The Bismuth system proposed before the advent of laparoscopic cholecystectomy divides biliary injury into five types (Table 1) (Fig. 6). Types 1 to 4 are based on the location of the injury in relation to the biliary ductal system. Injury to an aberrant right hepatic duct, which may or may not be associated with a common hepatic duct injury, constitutes a type 5 injury [35]. The Strasberg Classification, which is the most widely used, applies to biliary injuries encountered during laparoscopic cholecystectomy and is divided into types A to E (Table 2) (Fig. 6) [35, 36].

**Table 1:** Bismuth classification of biliary injuries during cholecystectomy

Bismuth classification of biliary injuries during cholecystectomy	
Type	Findings
1	Common hepatic duct or common bile duct injury greater than 2 cm away from the confluence of the right and left hepatic ducts
2	Common hepatic duct injury less than 2 cm away from the confluence of the right and left hepatic ducts
3	Hilar biliary injury with intact confluence of the right and left hepatic ducts
4	Hilar biliary injury with disruption of the confluence of the right and left hepatic ducts
5	Injury to an aberrant right hepatic duct associated with an injury to the common hepatic duct

**Table 2:** Strasberg classification of biliary injuries during cholecystectomy

Strasberg classification of biliary injuries during cholecystectomy	
Type	Findings
A	A leak arising from the cystic duct or from minor bile ducts in the surgical bed
B	Occlusion of a bile duct with proximal dilatation, most commonly involving an aberrant right hepatic duct
C	Transection without ligation of an aberrant right hepatic duct
D	A lateral injury involving a major bile duct
E	Bismuth type 1–5 injuries are classified as Strasberg type E1–E5 injuries

A patient with biliary injury may present with nonspecific symptoms such as abdominal pain, anorexia, and abdominal distention. If there is biliary obstruction, signs of jaundice will be present. In patients with a drain in place, bilious drainage is concerning for a bile leak [32, 37]. Postoperative liver function tests, which include serum bilirubin, alanine transaminase, aspartate transaminase, alkaline phosphatase, and gamma-glutamyl transpeptidase, should be obtained if there is suspicion of a bile duct injury [32, 33]. In case of biliary obstruction, serum bilirubin and cholestatic markers (alkaline phosphatase and gamma-glutamyl transpeptidase) are elevated. As the functioning of the liver is not impaired during the early stages, alanine transaminase and aspartate transaminase are usually not elevated [30, 32, 33].

**Fig. 7:**
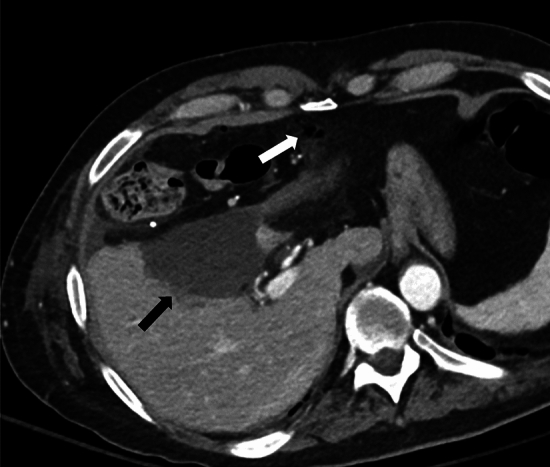
A 59-year-old who underwent cholecystectomy for acute on chronic cholecystitis with biloma secondary to bile leak. A postoperative collection is seen in the surgical bed (black arrow). Postsurgical pneumoperitoneum is also seen (white arrow). CT-guided aspiration revealed bilious contents. ERCP demonstrated a low-grade leak from the cystic duct, which was managed with biliary sphincterotomy and stent placement

**Fig. 8:**
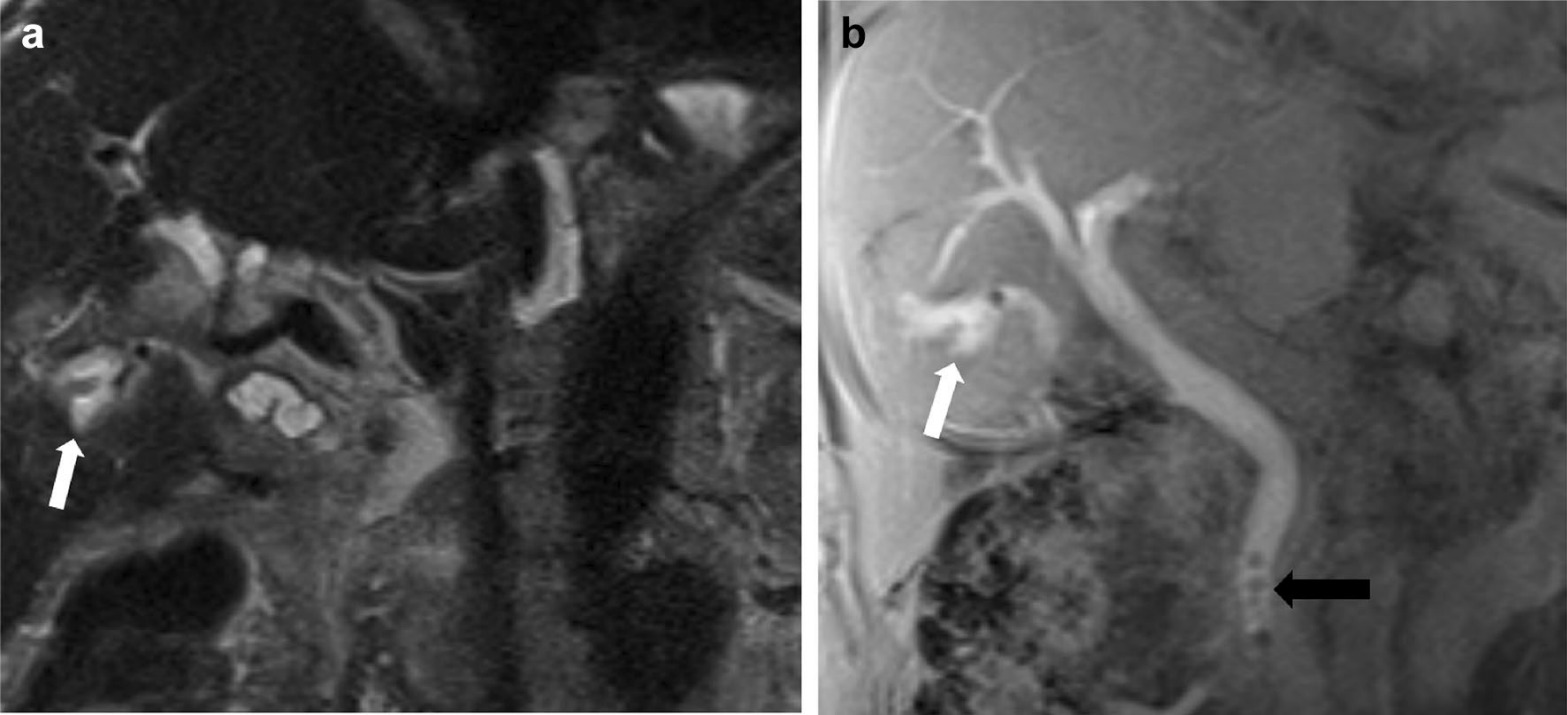
An 84-year-old male status post laparoscopic cholecystectomy for gallstone pancreatitis, with postoperative bile leak, confirmed using Eovist. A strong suspicion of a leak from the cystic duct stump was raised, which was confirmed on ERCP. **a** and **b** Coronal T2W image and 90 min delayed post-Eovist T1W image, respectively, showing a collection in gallbladder fossa with excretion of Eovist into the collection consistent with bile leak (white arrows). Pneumobilia in the terminal CBD (black arrow in image B) was better seen in a CT study performed earlier (not shown)

**Fig. 9:**
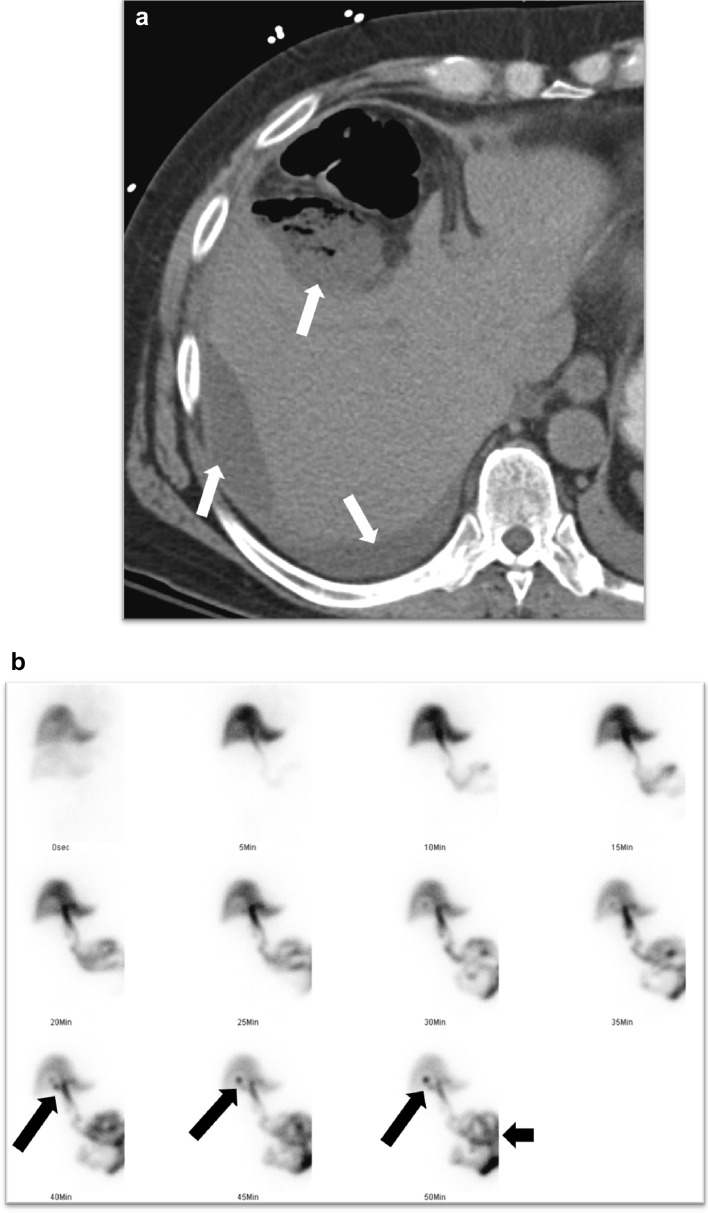
Bile leak post-cholecystectomy confirmed on a HIDA scan. **a** Axial CT image showing postsurgical collections in the gall bladder fossa and around the right lobe of the liver (white arrows). **b** The HIDA scan demonstrates progressive tracer accumulation in the gall bladder fossa, suggesting a bile leak (long black arrows). Tracer excretion into the bowel suggests maintained CBD continuity (short black arrow)

**Fig. 10:**
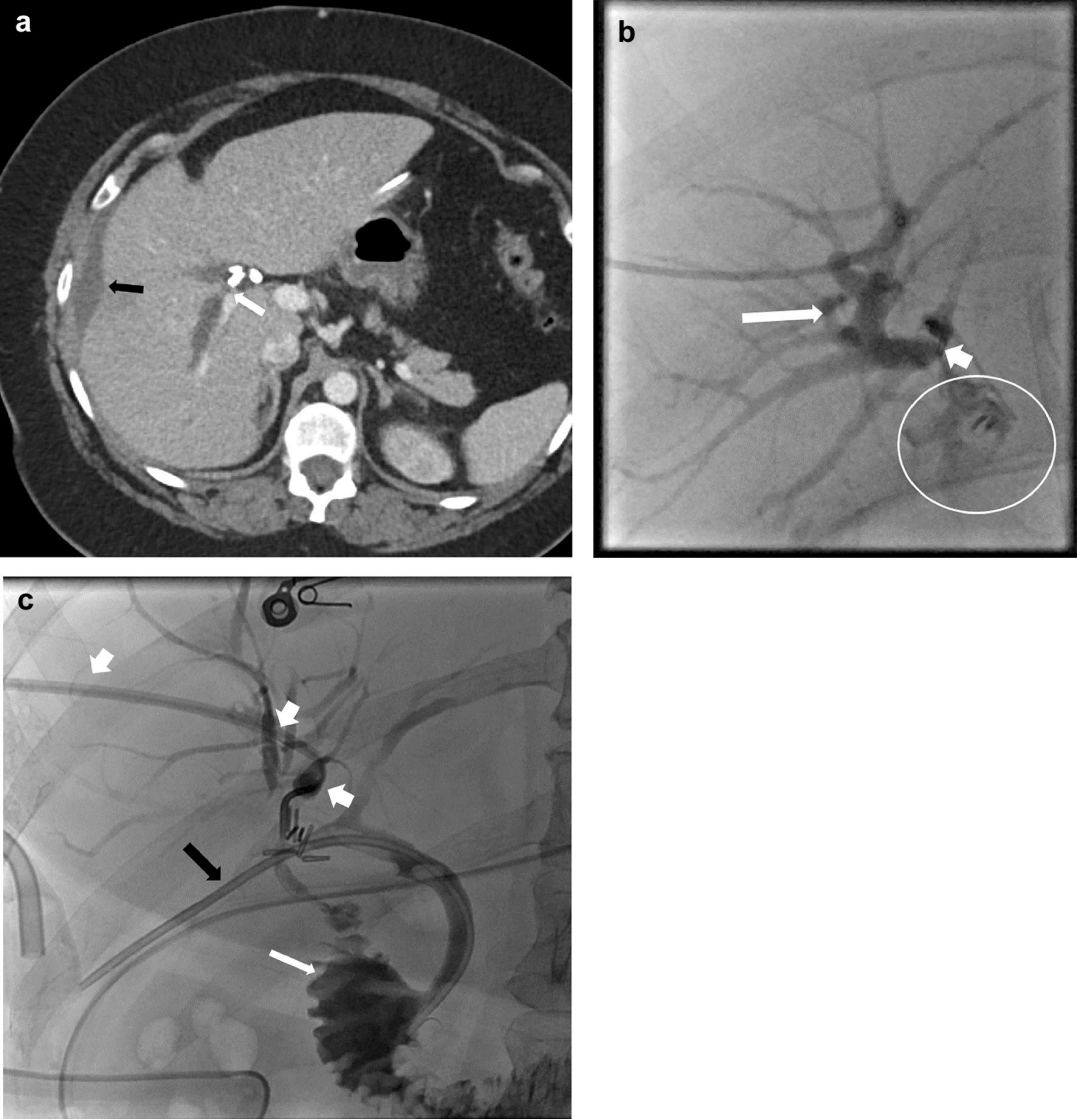
A 46-year-old female with right posterior hepatic duct injury, bile leak, and biliary obstruction involving the posterior segments of the right hepatic lobe. **a** axial CECT image demonstrating a biliary dilatation involving the right posterior hepatic lobe with abrupt narrowing adjacent to a surgical clip in the hepatic hilum (white arrow). Perihepatic fluid collection is also demonstrated (black arrow). MRI with Eovist contrast and MRCP also confirmed biliary dilatation in the right posterior hepatic lobe and leak of Eovist into the perihepatic collection (not shown). **b** PTC image demonstrating intrahepatic biliary dilatation (long white arrow) upstream to a surgical clip (short white arrow) and associated bile leak (white circle). **c** PTC post placement of internal/external bile drain (short white arrows) and recanalization of the occluded right posterior duct demonstrates the passage of the contrast into the duodenum (long white arrow). A common bile duct stent is also seen (black arrow)

In case of bile leak, ultrasound or CT will demonstrate a collection in the surgical bed or in the abdominal cavity but cannot ascertain bile leak as a source of these collections. Biliary dilatation may be evident if there is bile duct obstruction. These imaging modalities help perform guided drainage, and biochemical analysis of the fluid can confirm a bile leak. Biloma formation results from a localized collection of bile [8, 27] (Figs. 7, 8, 9 and 10).

**Fig. 11:**
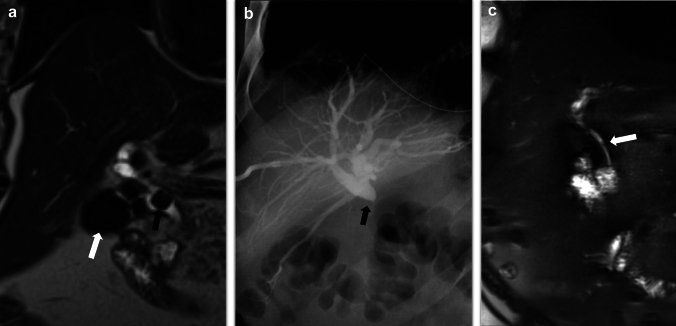
Common bile duct stricture formation. **a** Coronal MRCP image demonstrating cholelithiasis (white arrow) and a stone at the junction of the cystic duct to the CBD (black arrow). During the cholecystectomy, extensive adhesions were seen in the surgical bed, and a cholecysto-choledochal fistula was also seen. **b** T tube cholangiogram for postoperative jaundice shows a tight CBD stricture (black arrow) with non-opacification of the distal CBD. The stricture was managed by ERCP and stenting. **c** MRCP image 1-year post-procedure shows non-dilated CBD with focal residual luminal narrowing (white arrow)

**Fig. 12:**
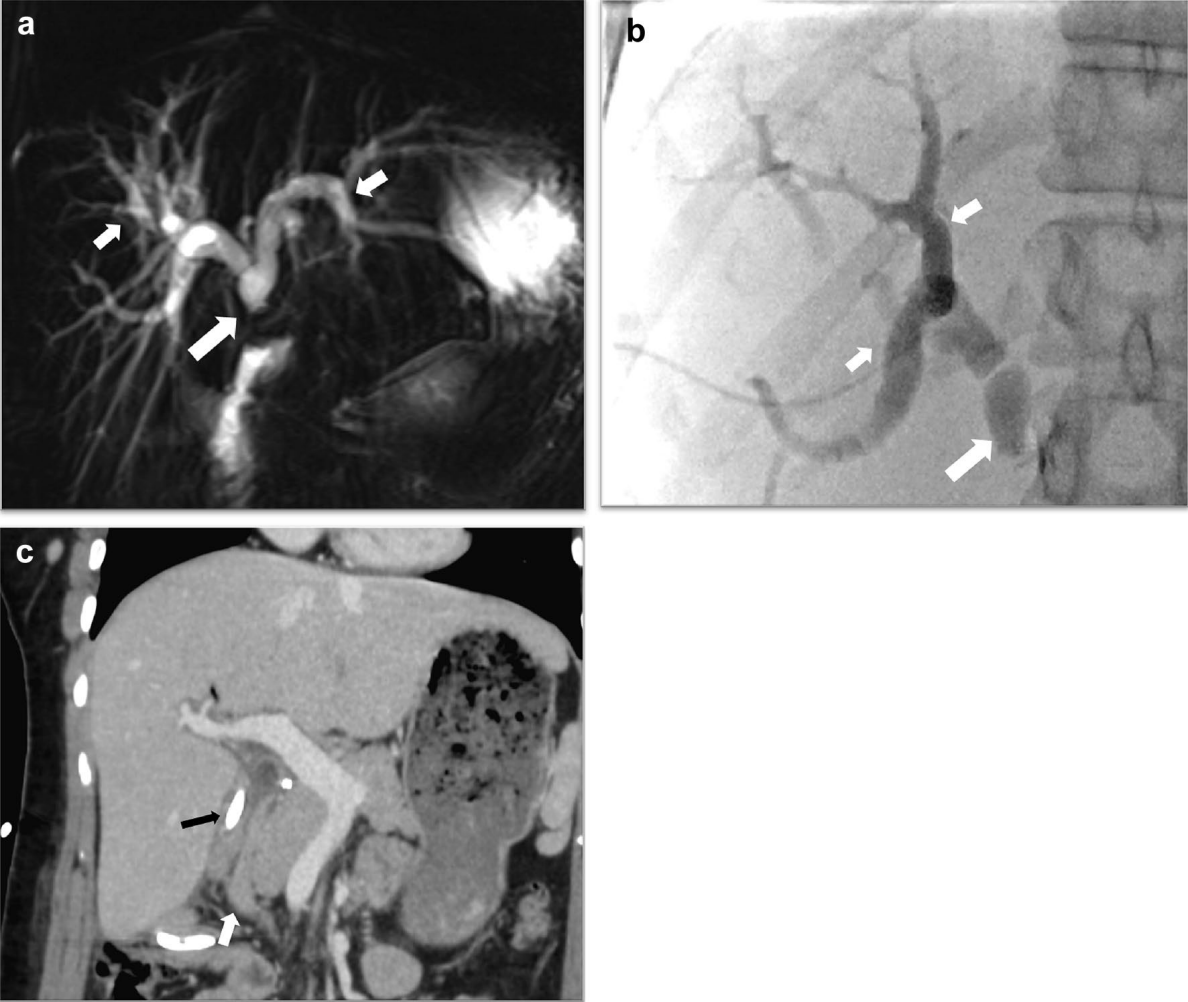
A 28-year-old female with transection of the CBD during cholecystectomy. She presented with jaundice 6 days post-cholecystectomy. **a** Coronal MRCP and **b** Percutaneous transhepatic cholangiography in the coronal plane demonstrate abrupt cut-off of the CBD (long white arrow) and biliary dilatation (short white arrows). **c)** Coronal CT image post hepaticojejunostomy with a Roux-en-Y jejunal loop, which shows a stent within it (black arrow) to the right of the duodenum (white arrow)

MRI with MRCP is the radiological investigation of choice for evaluating biliary anatomy and visualization of retained stones. Delayed T1-weighted MRI images post administration of hepatobiliary excreted contrast (Eovist) can help diagnose a bile leak, which will be seen as extraluminal leakage of contrast (Fig. 8). Further delayed imaging should be obtained if the leak is not apparent on the routinely obtained 20-min delayed T1-weighted sequence. Excretion of Eovist into the biliary system can be delayed in the setting of hepatic dysfunction [38]. In addition to extraluminal contrast extravasation, the site of the leak may also be identified, especially if the injury involves the larger ducts, which can then help with surgical planning [15, 27]. In cases of biliary obstruction or stricture formation, MRCP will demonstrate upstream dilatation and abrupt narrowing at the site of the pathology (Figs. 10, 11 and 12). If there is a complete bile duct transection, MRCP has an added advantage over ERCP for evaluating the biliary system upstream to the cut-off point [33] (Fig. 12).

A HIDA scan can also demonstrate bile leak as tracer accumulation in the gall bladder fossa, which progressively increases over time [7, 8] (Fig. 9b). SPECT-CT helps in anatomical localization and can also confirm the diagnosis if the findings are equivocal on the planar images. [26]. If biliary continuity is disrupted, tracer drainage into the small bowel will not be seen. Elevated bilirubin levels above 5 mg/dl may decrease the sensitivity of the examination [39].

ERCP and PTC are invasive modalities that help diagnose and manage biliary complications. They can identify the precise location of a bile leak or a biliary stricture, and biliary interventions like decompression and stent placement can be performed. ERCP can accurately identify a bile leak and is the first line in managing major bile duct injury. If the continuity of the ductal system is maintained, a stent can be placed across the site of injury to manage the leak. PTC can also characterize and treat biliary complications as an adjunct to ERCP, in cases of ERCP failure, or when ERCP cannot be performed, for example in Roux-en-Y gastric bypass surgery patients. As ERCP and PTC are invasive procedures, patients can develop complications, including post-ERCP pancreatitis and cholangitis [15, 34, 37].

Bile duct injuries can be treated with percutaneous drainage, ERCP, and PTC, either alone or in combination, depending upon the site of the leak and the disrupted duct, its severity, the time of recognition of the injury, and associated complications [30–32, 34, 37] (Figs. 10, 11). If non-surgical management fails, then surgical intervention needs to be performed. The injuries requiring surgical management include almost all cases of complete transection or ligation of the major bile ducts, large lateral injuries of the major ducts, and cases of biliary strictures when there is failure of nonsurgical management. A Roux-en-Y hepaticojejunostomy to the proximal bile duct stump is the preferred surgical method for reconstruction in most major biliary injuries [34] (Fig. 12c).

### Gallstone-related complications

**Fig. 13:**
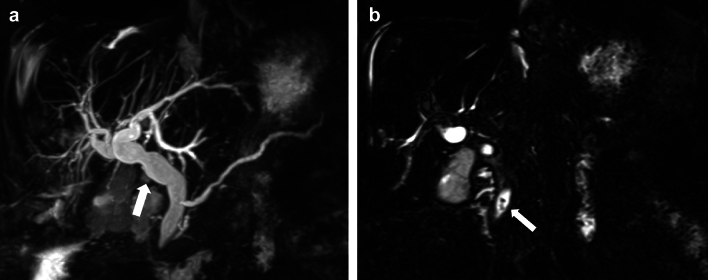
A 75-year-old female with retained gallstones post-cholecystectomy. MRCP abdomen was performed due to elevated liver function tests and an ultrasound of the abdomen showing worsening biliary dilatation. **a** and **b** Coronal MIP MRCP and Coronal 3D SPACE MRCP images show biliary dilatation (arrow in image a) and retained gallstones in the terminal common bile duct (arrow in image b)

Post-cholecystectomy, gallstones may be retained in the extrahepatic biliary tree at the time of the surgery or can form later. They can be diagnosed incidentally, or the patient may present with symptoms such as biliary colic, obstructive jaundice, acute pancreatitis, or cholangitis [40]. Serum alkaline phosphatase, a marker for cholestasis, can be elevated. Ultrasound of the right upper quadrant can demonstrate associated biliary dilatation and the stone(s) in the biliary system if the acoustic window is optimal. Radiopaque gallstones can be easily identified on CT. MRCP is the imaging modality of choice for the evaluation of choledocholithiasis. The stones are seen as signal voids in the biliary system on the T2-weighted sequences [7, 8] (Fig. 13b). They should be differentiated from other causes of a signal void, which can mimic stones and include foci of air, flow artifacts, extrinsic impressions by vessels, and a prominent duodenal papilla [41].

**Fig. 14:**
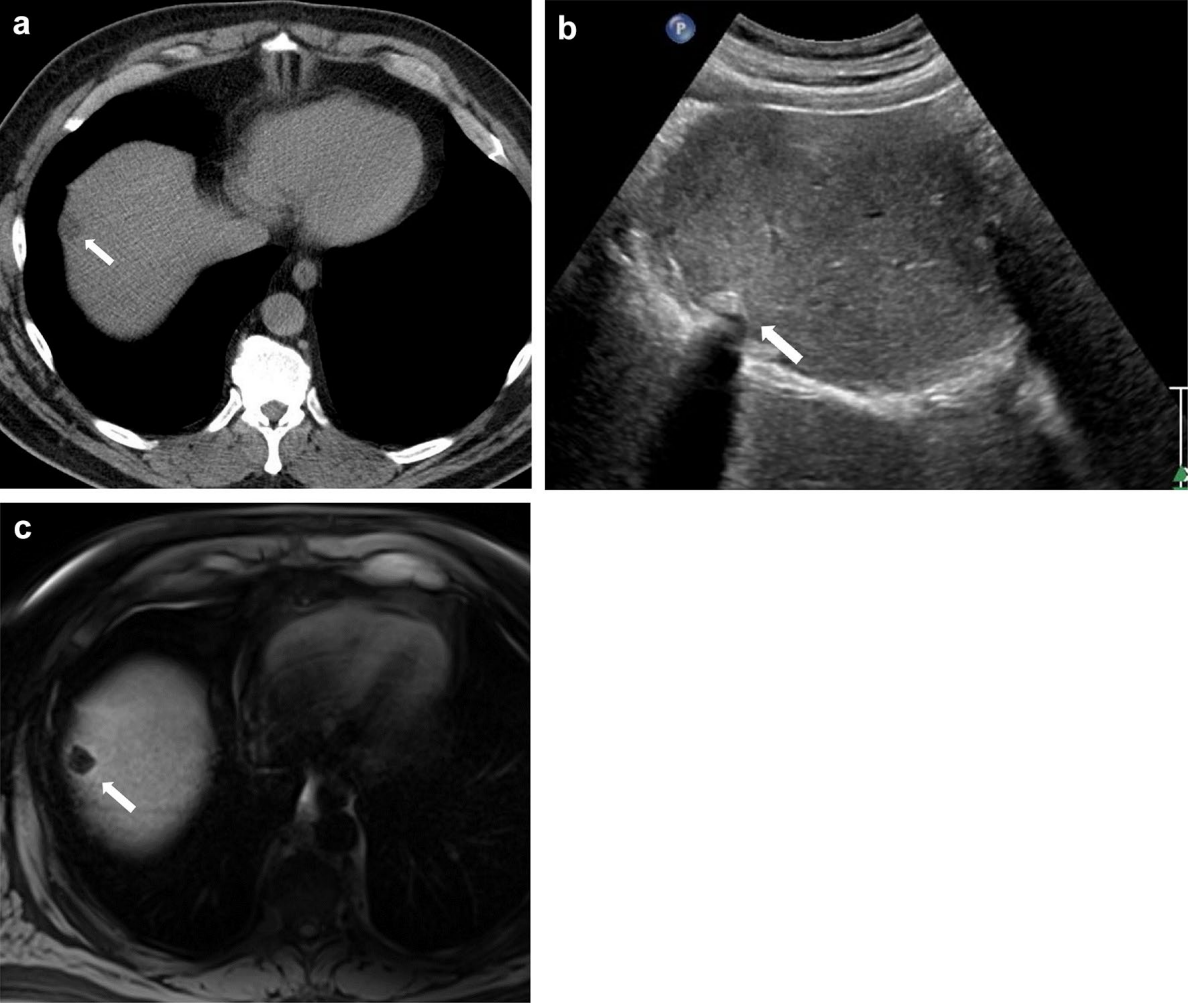
A 51-year-old male presented with dropped gallstones. He presented with intractable hiccups a few months post-cholecystectomy. A chest CT was performed for evaluation, which was unremarkable. However, an indeterminate lesion was seen in the right hepatic dome. **a** Axial noncontrast CT image demonstrating a hypodense lesion in the right hepatic dome (arrow). Ultrasound of the right upper quadrant was performed to further characterize this lesion. **b** An ultrasound image of the area of interest demonstrates a hyperechoic focus with posterior acoustic shadowing in the right subdiaphragmatic region indenting the right lobe of the liver, consistent with a dropped gallstone (arrow). **c)** T1-weighted MR image also demonstrates the dropped gallstone as a signal void (arrow). Multiple gallstones were seen in the subhepatic region and in the gallbladder fossa (not shown)

**Fig. 15:**
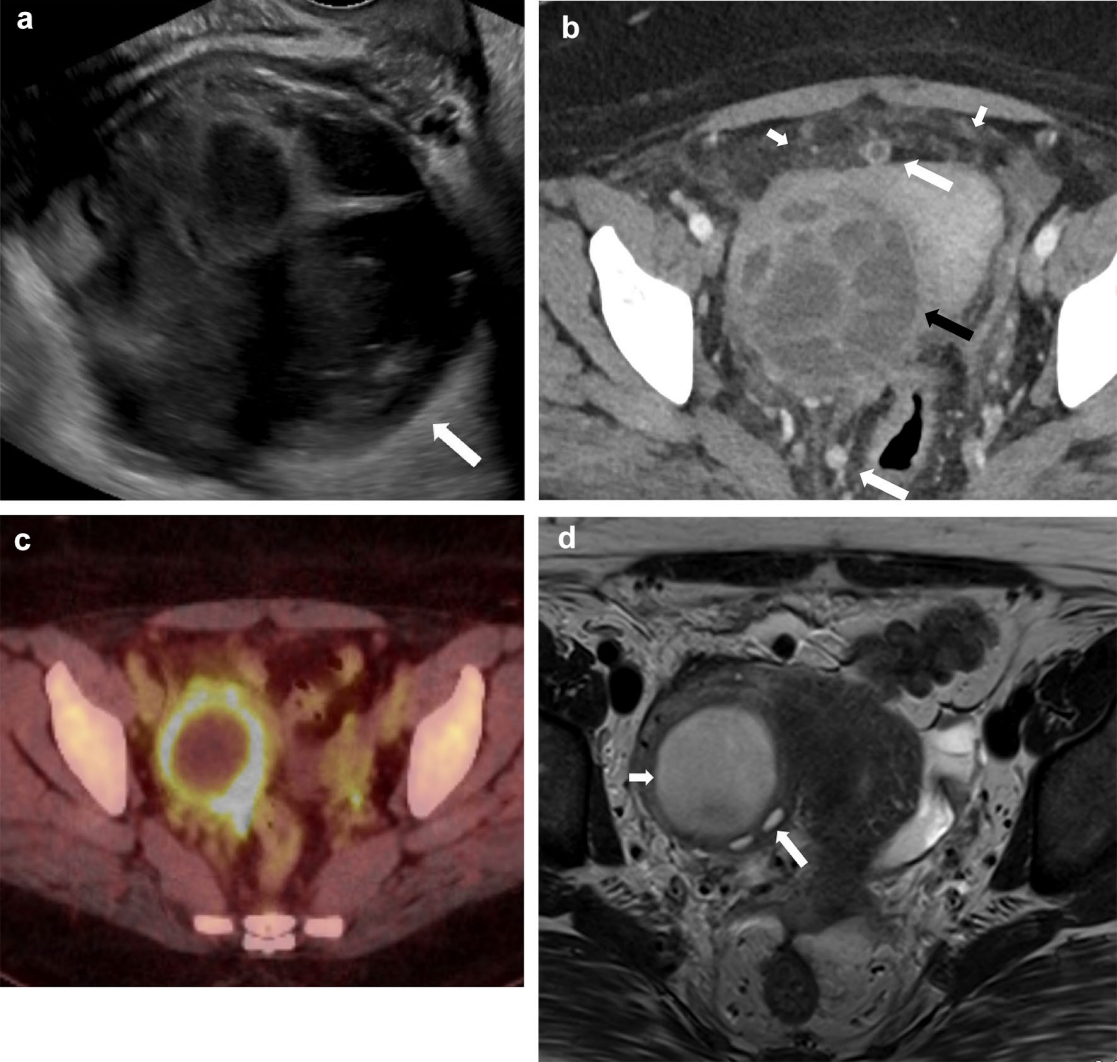
A 46-year-old female with dropped gallstones and a resultant pelvic abscess presenting as a pelvic mass. **a** Ultrasound of the pelvis showed a complex right adnexal mass (arrow). The right ovary was not visualized separately. CT abdomen pelvis was performed. **b** Axial CECT image showing a complex multiseptated right adnexal mass demonstrating loss of fat planes with the uterus (black arrow), associated fat stranding (short white arrows), and rounded peritoneal nodules (long white arrows). The patient was treated with antibiotics and underwent laparoscopy, but the procedure was aborted as the surgeon saw multiple peritoneal nodules in the pelvis. **c)** PET/CT was performed post laparoscopy, Axial PET/CT image shows uptake in the periphery of the mass (arrow). MRI of the abdomen was performed 2 weeks after the CT study. **d)** Axial T2 weighted MRI image showing interval decrease in loculations in the cyst (short arrow). The cyst is inseparable from the right ovary (long arrow). The patient underwent a hysterectomy, bilateral salpingectomy, and right oophorectomy. On pathology, the multiple peritoneal nodules seen on imaging were consistent with gallstones. The right adnexal mass showed inflammatory contents and golden-brown sediment within it, consistent with bile

Dropped gallstones are a common complication of laparoscopic cholecystectomy, with a reported incidence of 36% [42]. Gallstones can be dropped either during cholecystectomy or secondary to gallbladder perforation. This occurs when there is a breach in the gallbladder wall during the surgical procedure or acute inflammation. It may not be possible to remove the stones during the surgery, and up to 16–50% of the stones may be retained in the abdomen [43]. As this is a relatively common occurrence and not always recognized, surgeons may not always document spillage of gallstones as a complication of the surgical procedure. Furthermore, retained intraperitoneal gallstones are typically asymptomatic, and some may believe that their presence can lead to unnecessary anxiety in the patient [44]. Dropped gallstones may result in complications in 0.1–6% of the patients [45]. These stones are freely mobile in the abdominal cavity and can lodge in remote locations. Therefore, a high index of suspicion is required during imaging to diagnose complications of dropped gallstones. Gallstones will be seen as echogenic foci with posterior acoustic shadowing on ultrasound (Fig. 14b). CT can identify dropped gallstones if they are calcified. On MRI, they will be seen as signal voids (Fig. 14c). The dropped gallstones can serve as a nidus for infection, and the most common complications include intraabdominal, abdominal wall, and retroperitoneal abscesses. Abscesses constitute about 50–60% of the resultant complications (Fig. 15). Other reported complications include the formation of sinuses and granulomas, intestinal obstruction, and distant migration, including to the chest and urinary tract [43, 46]. Low phospholipid-associated cholelithiasis (LPAC) syndrome is a rare condition that is caused by ABCB4/MDR3 mutation. In this condition, there is low biliary phospholipid concentration, which results in symptomatic and recurring cholelithiasis, and intrahepatic lithiasis can develop post-cholecystectomy [47, 48].

### Vascular complications

**Fig. 16:**
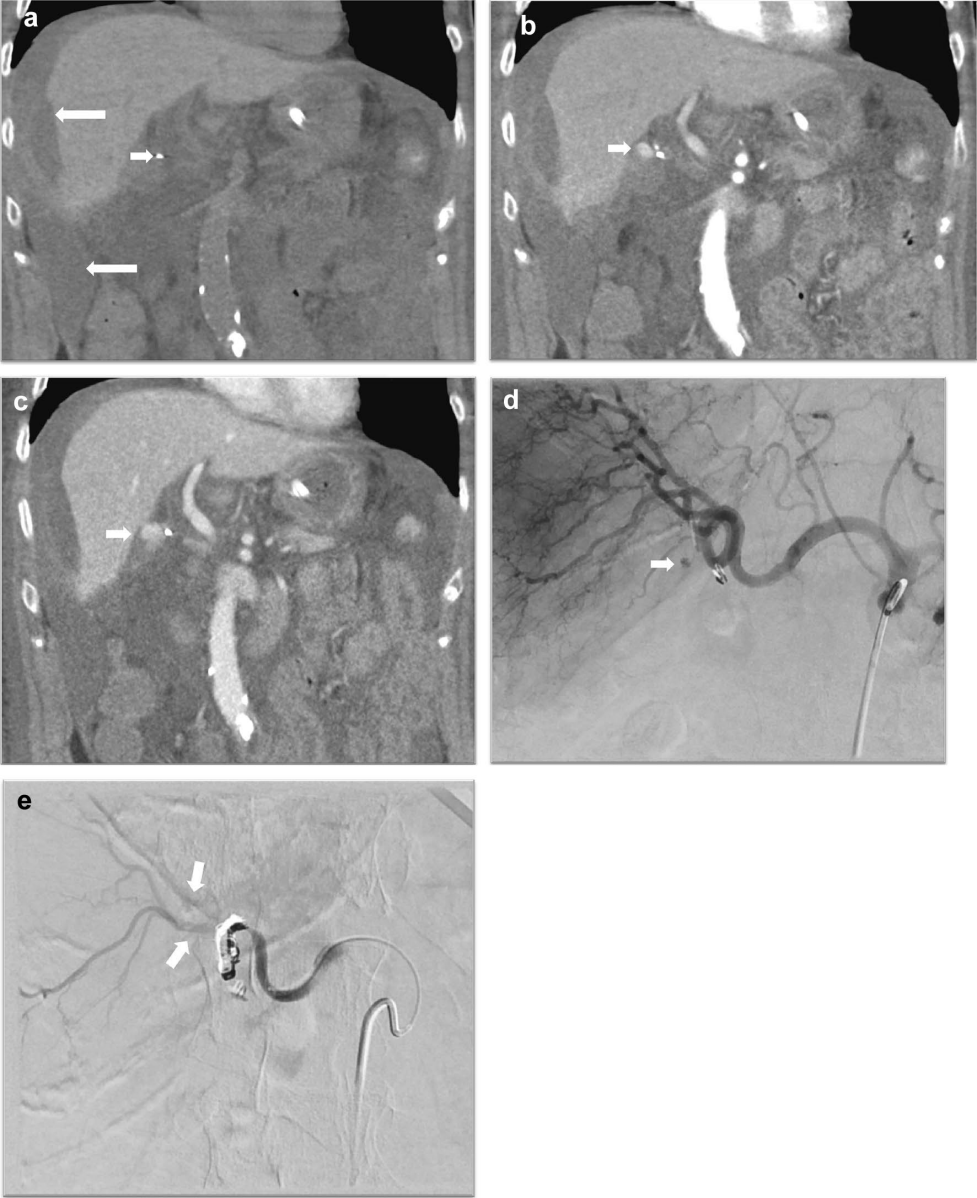
A 70-year-old male status post cholecystectomy, with bleeding from cystic artery stump. CT Abdomen was obtained 10 days post-cholecystectomy for down-trending hemoglobin. **a**, **b** and **c** Coronal non-contrast, arterial, and venous phase images, respectively, showing active expanding contrast extravasation (short arrows in images b and c) adjacent to a clip at the surgical site (short arrow in image a). Associated large hemoperitoneum (long arrows in image a). **d** The catheter celiac angiogram demonstrated a contrast blush adjacent to the clip, which was arising from the proximal right hepatic artery (arrow) in the region of the cystic artery stump. **e** Status post coil embolization of the proximal right hepatic artery with collateral flow opacifying the distal branches (arrows)

Vascular complications are rare during cholecystectomy. Factors that can predispose to vascular injuries include anatomical variants, difficult dissection, and patient-related factors such as obesity. The right hepatic artery is the most common vascular structure injured during cholecystectomy, followed by the portal vein [8]. The complications include active bleeding, pseudoaneurysm formation, and inadvertent vessel ligation or vascular thrombosis, which can lead to end-organ ischemia. Complications can also arise from the cystic artery stump in case of improper ligation or a slipped ligature, which can present as postoperative hemorrhage (Fig. 16). Active bleeding and pseudoaneurysm formation are best evaluated on CT angiography. In the case of arterial injury, active bleeding is seen as a blush or swirling of contrast on the arterial phase, with contrast pooling on the venous phase (Fig. 16). However, a pseudoaneurysm (with no associated active bleed) will not show a pooling of contrast on delayed images [26]. Injuries to the portal vein are rare and generally occur in association with injuries to the biliary system and the right hepatic artery. Patients with prominent middle hepatic vein branches running close to the gallbladder fossa are at risk for hemorrhage from the liver bed during cholecystectomy [17]. Epigastric vessels and, rarely, the aorta or the inferior vena cava may be injured during the insertion of the Veress’ needle or the first trocar during the creation of pneumoperitoneum [49, 50].

**Fig. 17:**
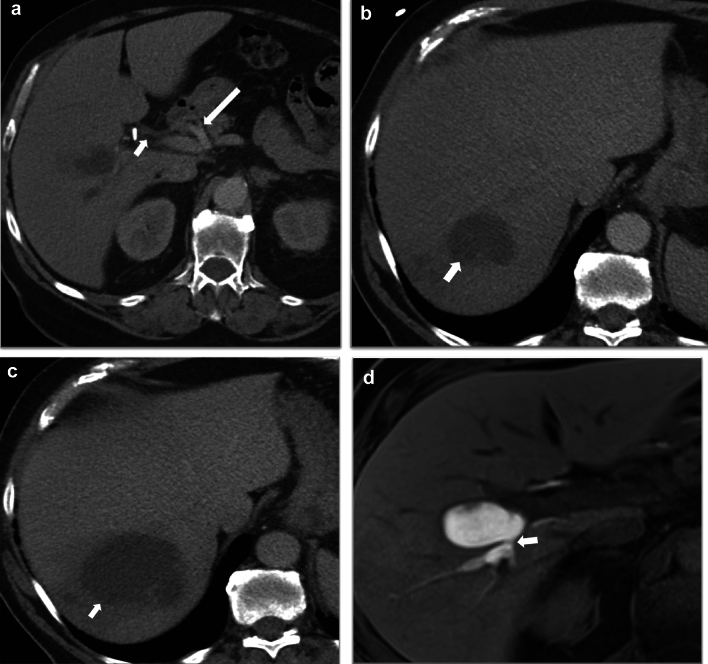
A 77-year-old male with right hepatic artery thrombosis post cholecystectomy leading to biliary ischemia in the right hepatic lobe and resulting in the formation of multiple bilomas. **a** Axial post-contrast CT demonstrating non-opacification of the right hepatic artery consistent with thrombosis (short arrow) and a surgical clip closely abutting it, likely representing inadvertent clipping of the artery and resultant thrombosis. The common hepatic artery (long arrow) and the left hepatic artery (not shown) were patent. **b** Axial CT post-contrast image shows a cystic lesion in the right hepatic lobe (arrow). Multiple such lesions were seen in the right lobe and were thought to be abscesses. **c** A follow-up CT performed after 1 month showed an increase in the interval size (arrow). Drain placement resulted in continuous bile drainage, confirming the diagnosis of bilomas. d) Axial post-Eovist MRI images show a biloma communicating with the biliary tree (arrow). No bilomas were seen in the left hepatic lobe. The cause for these multiple bilomas involving the right hepatic lobe was the right hepatic artery injury leading to biliary ischemia

Combined biliary and vascular injuries are termed vasculobiliary injuries. The vascular component of the injury can involve the hepatic arterial system and/or portal vein. The biliary component can be secondary to an operative complication or biliary ischemia [51]. Right hepatic artery injury is frequently associated with a biliary injury [17]. The hepatic artery and portal venous system perfuse the hepatic parenchyma by dual vascular supply. However, the biliary tree is exclusively supplied by the arterial system. If there is occlusion of the right hepatic artery alone, collateral supply via the arterial plexus along the biliary tree from the hepatic hilum generally compensates for the loss of supply from the right hepatic artery in the majority of cases, and it rarely causes clinical symptoms [17, 51, 52]. However, if collateral formation is inadequate, it can result in biliary ischemia (Fig. 17), hepatic necrosis, or parenchymal atrophy [17].

### Postsurgical collections

**Fig. 18:**
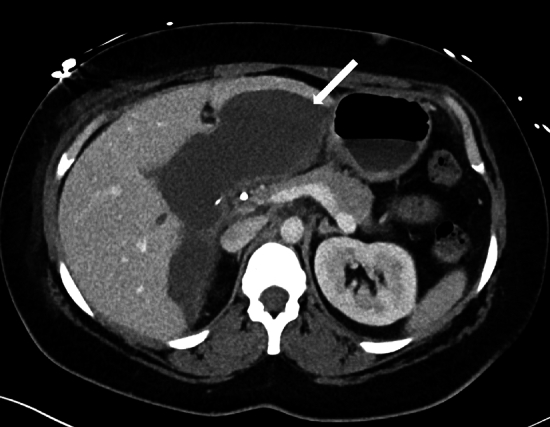
A 29-year-old female presenting with postoperative pain, tachycardia, and leukocytosis post cholecystectomy with subcapsular hepatic hematoma. An axial CECT image of the abdomen performed 2 weeks after surgery demonstrates a large subcapsular hepatic collection indenting the left hepatic lobe (arrow). Drainage of the collection showed serosanguinous fluid consistent with hematoma. An MRI of the abdomen using Eovist contrast was also performed, which did not show a bile leak (not shown)

Postsurgical hematoma typically presents as a high-density collection in the surgical bed. Acute bleeding demonstrates a density of about 30–45 Hounsfield units. In comparison, clotted blood demonstrates geographic areas of high attenuation with a density greater than 60 Hounsfield units, representing the clot surrounded by low-attenuation serum. In case of a nonlocalized bleed, a large clot (sentinel clot sign) may be present in the gallbladder fossa [53]. The density of a collection less than 30 Hounsfield units does not reliably exclude bleeding as an etiology, as liquefaction of the clot and dilution of the hyperdense bleed by reactive ascites may artefactually decrease the attenuation value of a collection [54] (Fig. 18). Hemorrhage identified on postoperative imaging can be due to bleeding from small arteries/veins in the surgical bed (which generally resolves spontaneously), slipped ligatures, inadequate hemostasis during surgery, or injury to the major vessels discussed in the previous section [49].

A biloma is a localized collection of bile that may or may not communicate with the biliary system [55]. It generally results from an injury to the biliary system. Imaging would show a low-attenuation collection generally seen in or close to the surgical bed and perihepatic regions, but it can also form elsewhere in the abdomen [55, 56]. Imaging confirmation can be obtained with a HIDA scan or MRI with hepatobiliary contrast agent. Aspiration can confirm diagnosis as fluid analysis will demonstrate increased bilirubin levels.

**Fig. 19:**
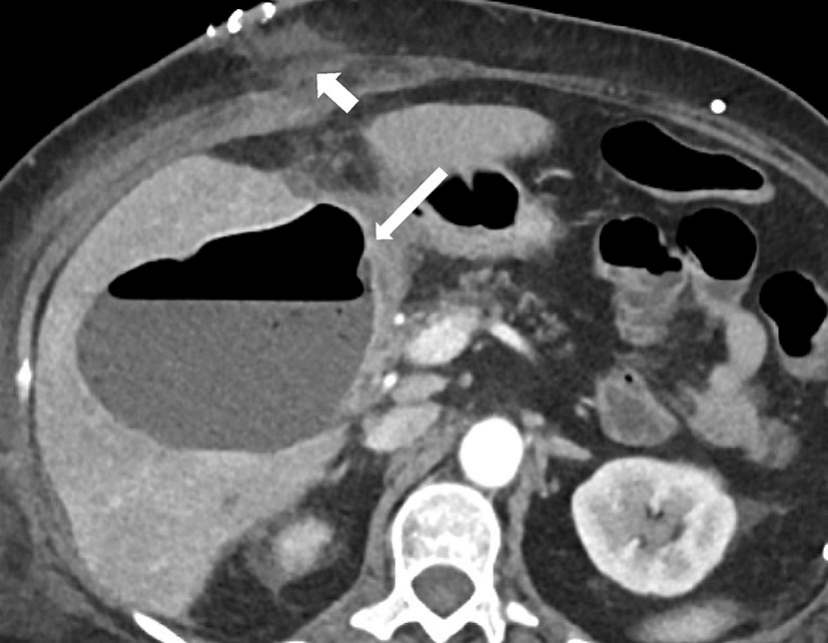
A 57-year-old woman with an abscess in the gallbladder fossa post-cholecystectomy. Axial postcontrast CT image demonstrates a thick-walled collection in the surgical bed, air-fluid level, and rim enhancement consistent with postsurgical abscess (long arrow). Postsurgical fat stranding and suture material are also seen in the anterior abdominal wall (short arrow)

**Fig. 20:**
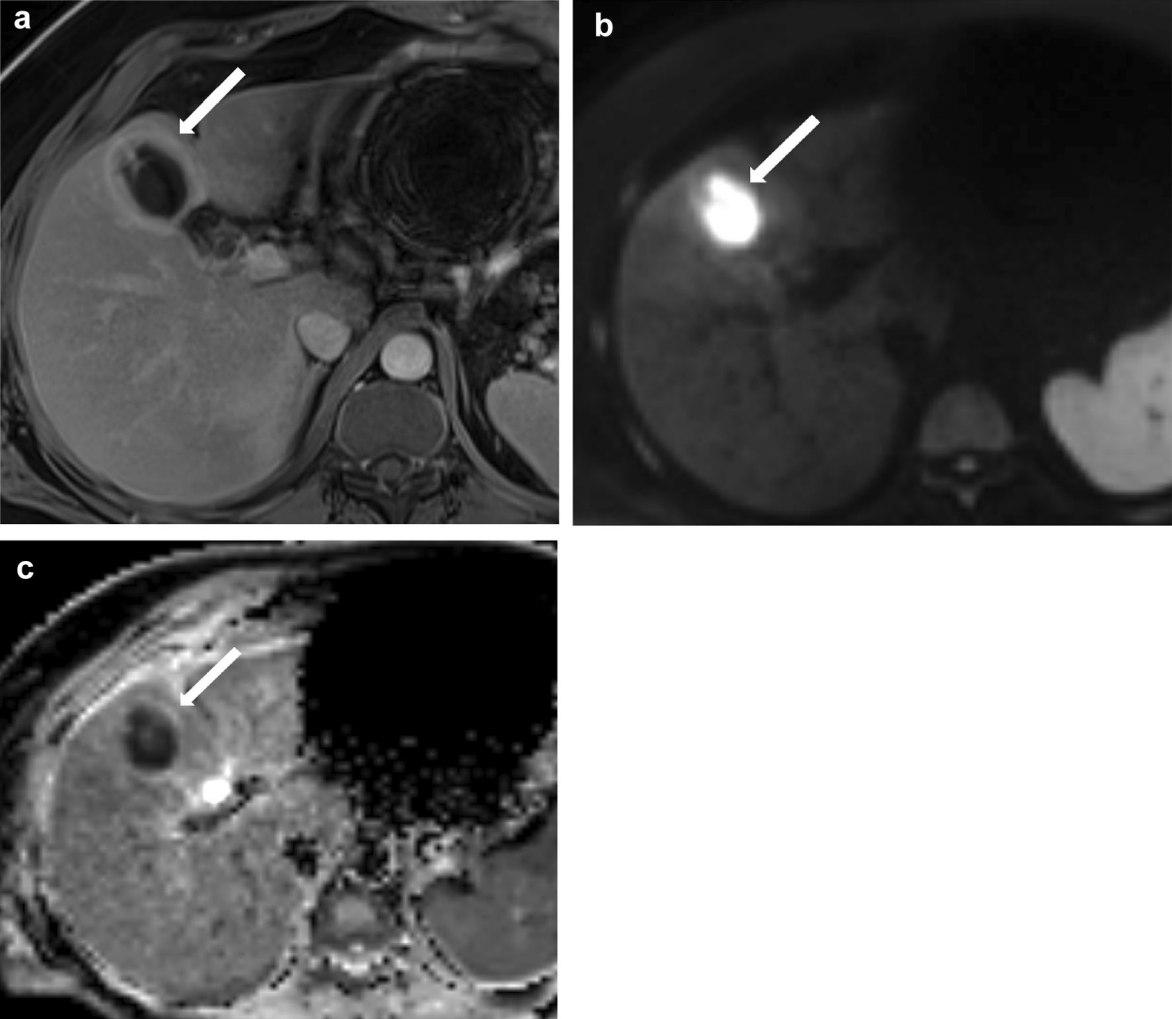
A 39-year-old male with hepatic abscess post-cholecystectomy. On surgery, it was found that the gallbladder had perforated into the adjacent liver. The patient had chronic right upper quadrant pain post-surgery. MRI Abdomen was performed 4 months post-surgery. **a**, **b** and **c** Axial post-contrast T1W image, DWI, and ADC images show a peripherally enhancing lesion demonstrating central diffusion restriction suggestive of a hepatic abscess (arrow). The abscess was drained under ultrasound guidance

An abscess typically presents as a rim-enhancing collection on CT and MRI (Fig. 19, 20). Diffusion restriction can be seen on MRI (Fig. 20). It may occur due to a preexisting infection in the surgical bed or can develop in a postsurgical collection, such as a biloma or a hematoma. The typical rim enhancement can be absent in the early phase, and at this stage, the patient's clinical presentation, laboratory investigations, and analysis of the aspirate help in the diagnosis [7, 8, 26].

### Other complications

#### Wound site complications

**Fig. 21:**
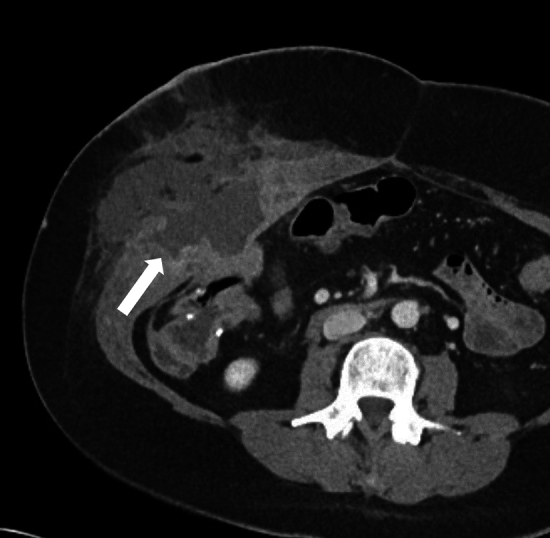
A 51-year-old female with an anterior abdominal wall abscess post-open cholecystectomy. Axial CECT shows a large collection (arrow) in the anterior abdominal wall at the incision site in the right upper quadrant with subtle rim enhancement and surrounding fat stranding

Wound site complications which can result from cholecystectomy are similar to those of other abdominal surgeries. Wound infections (Fig. 21) and hematomas can occur in the immediate postoperative period, with higher frequency in open cholecystectomy when compared to the laparoscopic route [5]. The imaging findings would be identical to hematomas or abscess formation in the gallbladder fossa, described in the previous section. A wound infection rate of 1.6% was reported post elective laparoscopic cholecystectomy, with increased body mass index identified as a risk factor [57].

Incisional hernias can develop in the anterior abdominal wall at the site of the incision in the right upper quadrant after open cholecystectomy and at the port sites after laparoscopic cholecystectomy. Factors that favor the development of incisional hernias after cholecystectomy include increased body mass index, the duration of the surgery, the diameter of the trocar, and the widening of the port for extracting the gallbladder [5].

#### Bowel injury

Bowel injuries during cholecystectomy can occur at the time of the first trocar insertion. If the patient has had prior abdominal surgery or has extensive pericholecystic inflammation, bowel adhesions may be present during the surgical procedure, which can increase the risk of bowel injury during the surgical procedure [5]. Inadvertent thermal injury to the bowel can also occur during cautery, by direct contact or through energy conduction and is rare [58]. The bowel injury may not be recognized at the time of the surgery. A high index of suspicion is required to diagnose this complication on imaging as postoperative pneumoperitoneum may be attributed to the recent surgery, and a bowel injury as an etiology may not be suspected [5]**.**

#### Clip migration

Surgical clip migration can occur post-cholecystectomy and is rare. The clips can get detached from the cystic duct stump and migrate in the abdominal cavity, similar to the mechanism of dropped gallstones. Clips can also migrate internally into the biliary tree. The migrated clips into the biliary tree can cause complications such as cholangitis or act as a nidus for stone formation [59, 60].

#### Incidental gall bladder *cancer*

**Fig. 22:**
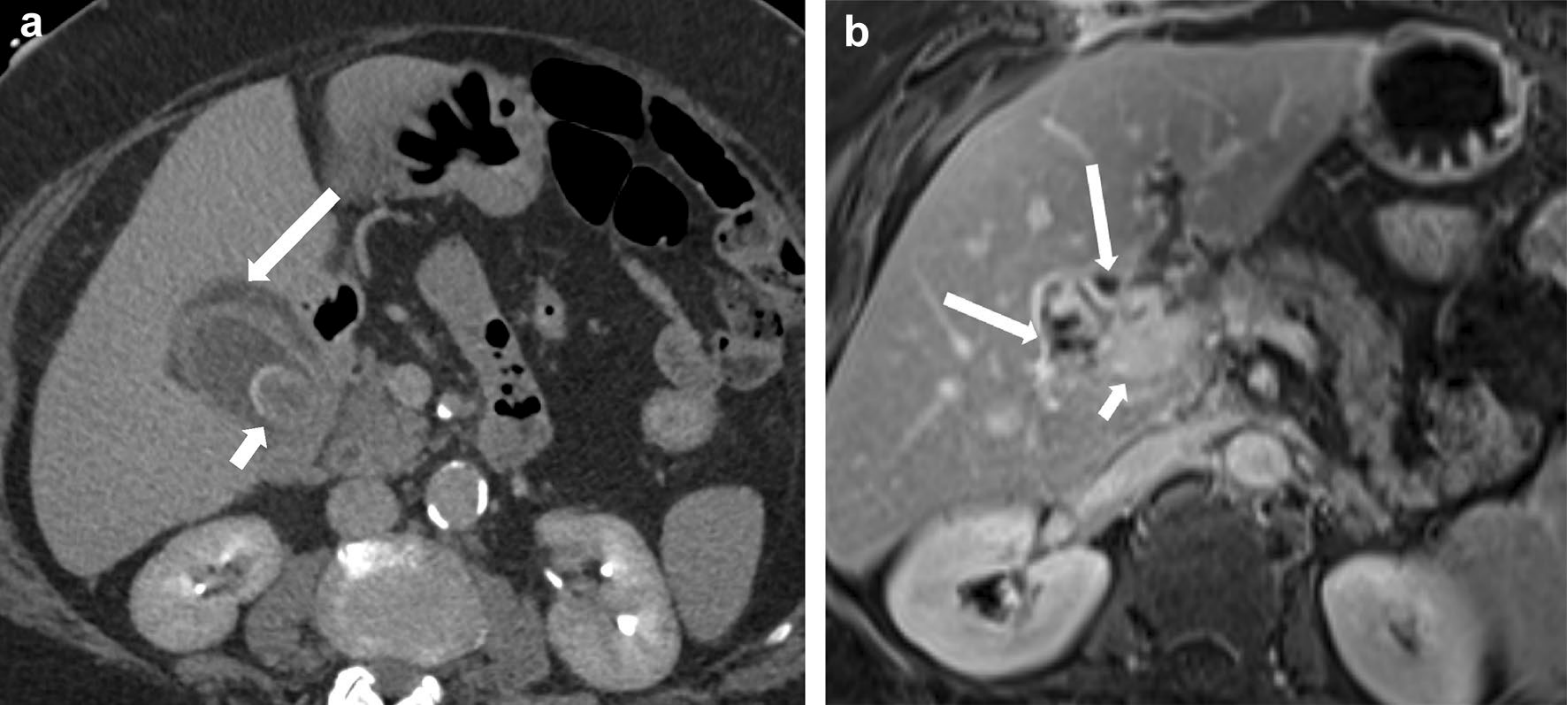
A 73-year-old female with gall bladder cancer diagnosed on pathology post cholecystectomy performed for acute cholecystitis. **a** Preoperative axial CECT showing cholelithiasis (short arrow) and gallbladder wall thickening (long arrow). Gallbladder carcinoma with periserosal infiltration and positive cystic duct margin was diagnosed on pathology. Central hepatectomy was planned after adjuvant chemotherapy. **b** Follow-up axial delayed post-contrast T1W image showing soft tissue (short arrow) encasing the intrahepatic and extrahepatic portal vein consistent with disease progression, with secondary biliary dilatation (long arrows)

Rarely gallbladder malignancy can be incidentally diagnosed on post-operative pathology specimens of cholecystectomies performed for benign indications [61] (Fig. 22). It has been reported that about 0.2–0.9% of the cholecystectomy specimens on pathological examination have an incidental diagnosis of gallbladder carcinoma [62]. If the pathology is confined to the mucosa, it has an excellent prognosis, and no further surgery is required. However, if this cancer has spread beyond the mucosa into the muscular layer of the gallbladder wall and beyond, restaging needs to be performed, and radical reresection is required if operable [61, 62]. It may be difficult to diagnose preoperatively due to the significant overlap in imaging appearance with acute or chronic cholecystitis when there is diffuse involvement of the gallbladder wall. Findings concerning gallbladder malignancy would include irregular, asymmetric wall thickening, irregular wall enhancement, absence of pericholecystic fat stranding, and fluid collections typically seen in acute cholecystitis [8].

## Conclusion

In conclusion, as the number of cholecystectomies continues to climb, the reliance on sophisticated imaging techniques will inevitably increase. The future of post-cholecystectomy care relies on meticulous surgical technique, vigilant postoperative monitoring, and the judicious use of imaging. Collaboration between surgeons and radiologists will continue to drive down complication rates and improve patient outcomes.
